# Effect of interaction between occupational stress and polymorphisms of MTHFR gene and SELE gene on hypertension

**DOI:** 10.7717/peerj.12914

**Published:** 2022-02-16

**Authors:** Fen Yang, Ruiying Qiu, Saimaitikari Abudoubari, Ning Tao, Hengqing An

**Affiliations:** 1 School of Public Health, Xinjiang Medical University, Urumqi Xinjiang, China; 2 The First Affiliated Hospital, Xinjiang Medical University, Urumqi Xinjiang, China; 3 Xinjiang Clinical Research Center for Genitourinary System, Urumqi Xinjiang, China

**Keywords:** Hypertension, Occupational stress, MTHFR gene, SELE gene, Interaction

## Abstract

**Background:**

Gene-environment interaction is related to the prevalence of hypertension, but the impact of genetic polymorphisms on hypertension may vary due to different geography and population.

**Objective:**

To explore the impact of the interaction among occupational stress and MTHFR gene and SELE gene polymorphism on the prevalence of hypertension in Xinjiang oil workers.

**Methods:**

A case-control study was conducted on 310 oil workers. In an oilfield base in Karamay City, Xinjiang, 155 hypertensive patients aged 18~60 years old with more than one year of service were selected as the case group, and 155 oil workers without hypertension were selected as the control group according to the 1:1 matching principle (matching conditions: the gender and shift were the same. The age is around 2 years old). The Occupational Stress Scale was used to evaluate the degree of occupational stress, PCR technique was used to detect MTHFR and SELE gene polymorphism, Logistic regression analysis was used to analyze the effects of gene and occupational stress on hypertension, and gene-gene and gene-environment interactions were analyzed by generalized multi-factor dimension reduction method.

**Results:**

The G98T polymorphism of SELE gene (*χ^2^* = 6.776, *P* = 0.034), the C677T (*χ^2^* = 7.130, *P* = 0.028) and A1298C (*χ^2^* = 12.036, *P* = 0.002) loci of MTHFR gene and the degree of occupational stress (*χ^2^* = 11.921, *P* = 0.003) were significantly different between the case group and the control group. The genotypes GT at the G98T polymorphism of the SELE gene (*OR* = 2.151, 95% CI [1.227–3.375]), and the dominant model (AC/CC *vs* AA, OR = 1.925, 95% CI [1.613–3.816]); AC and CC at the A1298C polymorphism of the MTHFR gene (*OR*_AC_ = 1.917, 95% CI [1.064–3.453]; *OR*_CC_ = 2.233, 95% CI [1.082–4.609]), the additive model (CC *vs* AA, OR = 2.497, 95% CI [1.277–4.883]) and the dominant model (AC/CC *vs* AA, OR = 2.012, 95% CI [1.200–3.373]); at the C677T polymorphism of the MTHFR gene CT and TT (*OR*_CT_ = 1.913, 95% CI [1.085–3.375]; *OR*_TT_ = 3.117, 95% CI [1.430–6.795]), the additive model (CC *vs* AA, OR = 1.913, 95% CI [1.085–3.375]) and the dominant model (AC/CC *vs* AA, OR = 2.012, 95% CI [1.200–3.373]), which could increase hypertension risk (*P* < 0.05). The gene-gene interaction showed that there was a positive interaction between the A1298C and C677T sites of the MTHFR gene, and the gene-occupational stress interaction showed that there was a positive interaction between the A1298C and C677T sites of the MTHFR gene and the occupational stress.

**Conclusion:**

The interaction of gene mutation and occupational stress in Xinjiang oil workers maybe increase the risk of hypertension.

## Introduction

High systolic blood pressure is a leading cause of death from ischemic heart disease, found in Global Burden of Disease Study 2019 (GBD 2019 Risk Factors Collaborators, 2019). Therefore, the effective prevention and control of hypertension in the population has become a major public health issue. Data from a cardiovascular disease risk screening study of 170,000 urban and rural residents showed that the detection rate of age-specific hypertension in people aged 35–75 was as high as 37% (Lu et al., 2017). However, the etiology of hypertension is complex and diverse, and has not yet been comprehensively studied. It has been shown that genetic and environmental interactions result in an increased risk of developing hypertension (Huang et al., 2019; Kokubo et al., 2019). Heritability studies have confirmed that genetic variation among individuals with hypertension accounts for approximately 30–60% of the variation (Liu et al., 2015). In recent years, a number of investigators have focused on the association of *MTHFR* gene (McNulty et al., 2017) *SELE* gene (Liao et al., 2016) polymorphisms with hypertension, but the findings have been inconsistent. Evidence from both the GWAS (Lu et al., 2015) and epidemiological studies (Amenyah et al., 2020; Ji et al., 2019) suggests that MTHFR gene polymorphisms are associated with the risk of developing hypertension, but polymorphisms at the C677T and A1298C loci of the MTHFR gene are not consistent in all populations. A case-control study in Orléans found that the prevalence of hypertension in the Algerian population was not associated with the MTHFR gene (Amrani-Midoun et al., 2016). Many studies have also confirmed that subjects carrying the 677CC genotype are at significantly increased risk of developing hypertension, while the C677T gene polymorphism leads to lower blood pressure (Ghogomu et al., 2016). Meta-analysis suggests that carriers of the C allele of the A561C polymorphism of the SELE gene may contribute to an increased risk of hypertension in the Chinese Han population (Ouyang et al., 2015). In addition, a significant association between G98T polymorphism and hypertension has also been found (Chen et al., 2008). From this it is clear that the probability of developing cardiovascular risk varies geographically due to genetic polymorphisms. Oil workers in Karamay, Xinjiang, are often associated with the occurrence of occupational stress due to the nature of their special occupation, which in turn affects their physical and mental health. A number of studies have now shown that occupational stress may cause an increase in blood pressure through the neurological response of individuals (Tao et al., 2018).

Therefore, considering the variation in the results of studies conducted in different populations, this study will comprehensively investigate the effects of the interaction among MTHFR gene and SELE gene polymorphisms and occupational stress on hypertension, that aim to provide new ideas for the prevention and control of hypertension in oil workers.

## Materials and Methods

### Study subjects

In the case-control study, a total of 183 oil workers with hypertension according to the results of physical examination in 2020 at the central hospital in Karamay, excluding 28 people with incomplete information (missing questionnaire information, no blood samples, low DNA extraction concentration, etc.), and finally including 155 hypertensive patients aged between 18–60 years with 1 year of work experience as the case group, and 155 non-hypertensive patients were selected as the control group according to the 1:1 matching principle with gender, age (±2 years), and shift status as matching factors. All participants signed a written informed consent. The study was approved by the Ethics Review Committee of the First Affiliated Hospital of Xinjiang Medical University (No. 2015006).

## Methods

### General information and occupational stress survey

A structured questionnaire was used to collect information on gender, age, educational level, shift work, smoking and alcohol consumption (Smoker: smoking ≥1 cigarette per day for six months or more; drinker: drinking ≥2 times a week with alcohol intake ≥50 g per drinking session regularly ≥1 year (Xing et al., 2018)), marital status, working age, personal income per month and BMI among oil workers. Among them, the Occupational Stress Inventory Revised Edition (OSI-R) was used to assess the level of occupational stress. The scale consists of the Occupational Role Questionnaire (ORQ), the Personal Strain Questionnaire (PSQ), and the Personal Resources Questionnaire (PRQ). The occupational stress of oil workers in this study was assessed by the ORQ, which consists of six dimensions with 10 entries in one dimension and using a scale of 1 to 5 and summarized, with higher ORQ scores representing higher levels of occupational stress. According to the scoring principle of the scale, occupational stress was classified as: “Low” (ORQ > 160), “Middle” (120 ≤ ORQ ≤ 160), and “High” (ORQ < 120).

### Diagnosis of hypertension and diagnostic criteria

Blood pressure was measured by a professional physician at the Karamay Central Hospital during a routine physical examination of the oil workers. After the oil workers rested for 10 min, blood pressure was measured twice at 5 min intervals using a zeroed standard mercury sphygmomanometer to take the mean value, and when the difference was greater than 5 mmHg, the mean value was taken after the third measurement. In this study, hypertension was defined according to the Chinese Guidelines for the Prevention and Treatment of Hypertension (2018 revision) (Liu, 2019) as (1) systolic blood pressure (SBP) ≥140 mmHg and/or diastolic blood pressure (DBP) ≥90 mmHg and (2) self-reported hypertension diagnosed by a physician, and being treated with antihypertensive therapy within the past two weeks.

### Sample collection

A total of 4 ml venous blood sample was collected from each subject after 8 h fasting period. The samples were put into the ethylenediaminetetraacetic acid (EDTA) anticoagulation tubes. The serum and plasma were separated by centrifugation at 4 °C, 8 000 r/min (20 mm radius), for 3 min, and then stored at −20 °C for future and used to extract the genomic deoxyribonucleic acids (DNAs) and ribonucleic acids (RNAs).

### Genotyping method of MTHFR and SELE gene polymorphisms

DNA in blood was quantified using spectrophotometric analysis, and a polymerase chain reaction-restriction fragment length polymorphism (PCR-RELP) technique to amplify the target fragment and digest the PCR amplified product with specific endonucleases for SNP typing, As follow:

First, the genomic DNA was extracted using a TIANamp Blood DNA Kit (TIANGEN, China) in accordance with the manufacturer’s instructions. Microspectrophotometer was used to detect the concentration and purity of the DNA samples for the subsequent experiments. Concentration >100 μg/μl, 1.70 <260OD/280OD <2.0 means that the sample is qualified. Second, all DNA samples were genotyped through polymerase chain reaction (PCR)–ligase detection reaction. Table 1 presents the primers and restriction endonuclease for amplifying the PCR of the target SNPs for each participant.

**Table 1:** The primers and restriction endonuclease for amplifying the PCR of the target SNPs.

	SELE A 561C	SELE G98T	MTHFR C677T	MTHFR A1298C
Primer sequences	F:ATTAGCATCAAGGTTTAGGATAGGT R:TGAAGAAAGAGAGGCAAGAACCA	F:TGCCCAAAATCTTAGGATGC R:AAGCCCAGGGAAGAACACAT	F:TGAAGGAGAGGTGTCTGCGGGA R:AGGACGGTGCGGTGAGAGTG	F:CTTCTACCTGAAGAGCAAGTC R:CATGTCCACAGCATGGAG
Length	326 bp	332 bp	198 bp	256 bp
REE (μl)	PstI(0.2)	HphI(1)	HinfI(1)	MboII(1)
10x Buffer (μl)	2	2	2	2
ddH2O (μl)	3	8.5	7	7
PCR products	17	8.5	10	10
Temperature (°C)	37	37	37	37
Time (min)	15	240	10	60
Agarose gels	2.5%	2.5%	3%	3%
Voltage	110 V	100 V	110 V	100 V
Genotyping	CC:326 bp	TT:332 bp	CC:198 bp	CC256 bp
	AA:222+104 bp	GG:194+138 bp	TT:175+23 bp	AA:17+22+30+27 bp
	AC:326+222+104 bp	GT:332+194+138 bp	CT:198+175+23 bp	AC:256+176+22+30+27 bp

### Statistical analysis

Comparison of rates and genotypes between groups for each polymorphism were performed using chi-square tests, all in SPSS 25.0. SHEsis software was used for Hardy-Weinberg equilibrium test. The effect of each gene polymorphism and occupational tension on hypertension was analyzed by logistic regression. Gene-gene and gene-occupational tension interactions were modeled by applying generalized multifactor downscaling (GMDR) software, and dendrograms were drawn with Multifactor dimensionality reduction (MDR) method.

## Results

### Distribution of basic demographic characteristics of the case and control groups

In this study, 310 oil workers (155 in the case group and 155 in the control group) were investigated, of whom 200 were male and 110 were female in Table 2. The comparison of hypertension among demographic characteristics showed that the differences between different personal income per month (*χ*^*2*^ = 5.684, *P* = 0.017), smoking (*χ*^*2*^ = 27.01, *P* < 0.001), drinking alcohol (*χ*^*2*^ = 8.903, *P* = 0.003), and BMI (*χ*^*2*^ = 5.845, *P* = 0.016) were statistically significant, while the differences between different ethnic group, educational level, professional title, marital status, and working age groups were not statistically significant (*P* > 0.05).

**Table 2:** Distribution of basic demographic characteristics of case group and control group.

Item	*N*	Case group		Control group		*χ* ^*2*^	*P*
		*n*	%	*n*	%		
Gender						0.000	1.000
Male	200	100	50.0	100	50.0		
Female	110	55	50.0	55	50.0		
Age/years	310	46.20 ± 5.458		46.21 ± 5.469		0.010	0.992
Shift work						0.000	1.000
Yes	216	108	50.0	108	50.0		
No	94	47	50.0	47	50.0		
Ethnic group						1.741	0.187
Han	253	122	48.2	131	51.8		
Others	57	33	57.9	24	42.1		
Educational level						3.780	0.052
High school or below	173	95	549	78	45.1		
College or above	137	60	43.8	77	56.2		
Professional title						1.229	0.268
Junior or below	95	43	45.3	52	54.7		
Intermediate or above	215	112	52.1	103	47.9		
Marital status						1.303	0.521
Single	13	8	61.5	5	38.5		
Married	259	126	48.6	133	51.4		
Divorced/others	38	21	55.3	17	44.7		
Personal income per month/Yuan						5.684	0.017
<5,000	202	111	55.0	91	945.0		
≥5,000	108	44	40.7	64	59.3		
Smoking						27.01	<0.001
Yes	183	114	62.3	69	37.7		
No	127	41	32.3	86	67.7		
Drinking alcohol						8.903	0.003
Yes	218	121	55.5	97	44.5		
No	92	34	37.0	58	63.0		
BMI (Body mass index/kg·m^−2^)						5.845	0.016
18.5~24	102	41	40.2	61	59.8		
>24	208	114	54.8	94	45.2		
Working age/years						1.685	0.194
≤15	113	51	45.1	62	54.9		
>15	197	104	52.8	93	47.2		
Total	310	155	50.0	155	50.0		

### Distribution of SELE and MTHFR genotypic loci and occupational stress between the case group and control group

The distribution of SELE and MTHFR genotype loci and occupational stress degree between the case group and the control group is shown in Table 3. Hardy-Weinberg genetic balance test showed that the distribution of G98T and A561C loci of SELE gene, A1298C and C677T loci of MTHFR gene were consistent with expected values in case group and control group. The G98T polymorphism of SELE gene (*χ*^*2*^ = 6.776, *P* = 0.034), the C677T (*χ*^*2*^ = 7.130, *P* = 0.028) and A1298C (*χ*^*2*^ = 12.036, *P* = 0.002) loci of MTHFR gene and the degree of occupational stress (*χ*^*2*^ = 11.921, *P* = 0.003) were significantly different between the case group and the control group.

**Table 3:** Distribution of gene and occupational stress between case group and control group.

Item	*N*	Case group		Control group		*χ* ^*2*^	*P*
		*n*	*%*	*n*	*%*		
*SELE* A561C						1.025	0.599
AA	213	103	48.4	110	51.6		
CC	15	9	60.0	6	40.0		
AC	82	43	52.4	39	47.6		
*SELE* G98T						6.776	0.034
GG	196	87	44.4	109	55.6		
TT	16	10	62.5	6	37.5		
GT	98	58	59.2	40	40.8		
*MTHFR* A1298C						12.036	0.002
AA	102	37	36.3	65	63.7		
CC	66	40	60.6	26	39.4		
AC	142	78	54.9	64	45.1		
*MTHFR* C677T						7.130	0.028
CC	116	50	43.1	66	56.9		
TT	52	34	65.3	18	34.6		
CT	142	71	50.0	71	50.0		
Occupational stress						11.921	0.003
Low	23	8	34.8	15	65.2		
Middle	127	52	40.9	75	59.1		
High	160	95	59.4	65	40.6		

### Logistic regression analysis of the relationship between genetic and occupational stress with hypertension

The association of SELE and MTHFR genes and occupational stress with hypertension was analyzed using binary logistic regression and is shown in Table 4. After adjusting for personal income per month, smoking, drinking alcohol, and BMI, genotypes GT at the G98T polymorphism of the SELE gene (OR = 2.151, 95% CI [1.227–3.375]), and the dominant model (AC/CC *vs* AA, OR = 1.925, 95% CI [1.613–3.816]); AC and CC at the A1298C polymorphism of the MTHFR gene (*OR*_AC_ = 1.917, 95% CI [1.064–3.453]; *OR*_CC_ = 2.233, 95% CI [1.082–4.609]), the additive model (CC *vs* AA, OR = 2.497, 95% CI [1.277–4.883]) and the dominant model(AC/CC *vs* AA, OR = 2.012, 95% CI [1.200–3.373]); at the C677T polymorphism of the MTHFR gene CT and TT (*OR*_CT_ = 1.913, 95% CI [1.085–3.375]; *OR*_TT_ = 3.117, 95% CI [1.430–6.795]), the additive model (CC *vs* AA, OR = 1.913, 95% CI [1.085–3.375]) and the dominant model(AC/CC *vs* AA, OR = 2.012, 95% CI [1.200–3.373]), which could increase hypertension risk (*P* < 0.05).

**Table 4:** logistic regression analysis of the influence of genes and occupational stress on the prevalence of hypertension.

Gene	Unadjusted		Adjusted	
	OR (95% CI)	*P*	OR (95% CI)	*P*
SELE A561C				
AA	1.00		1.00	
AC	1.073 [0.624–1.844]	0.800	1.161 [0.654–2.058]	0.610
CC	1.519 [0.496–4.652]	0.465	1.667 [0.495–5.617]	0.409
Additive	1.751 [0.547–5.601]	0.345	1.602 [0.551–4.658]	0.387
Dominant	1.243 [0.763–1.997]	0.392	1.337 [0.800–2.235]	0.268
Recessive	0.653 [0.227–1.882]	0.430	0.603 [0.192–1.896]	0.387
SELE G98T				
GG	1.00		1.00	
GT	2.039 [1.211–3.433]	0.007	2.151 [1.227–3.375]	0.008
TT	2.756 [0.924–8.216]	0.069	2.724 [0.879–8.439]	0.082
Additive	2.088 [0.730–5.971]	0.170	2.086 [0.666–6.535]	0.207
Dominant	1.852 [1.159–2.959]	0.010	1.925 [1.163–3.186]	0.011
Recessive	0.584 [0.207–1.648]	0.309	0.602 [0.199–1.822]	0.369
MTHFR A1298C				
AA	1.00		1.00	
AC	2.223 [1.279–3.864]	0.005	1.917 [1.064–3.453]	0.030
CC	2.682 [1.351–5.327]	0.005	2.233 [1.082–4.609]	0.030
Additive	2.703 [1.428–5.114]	0.002	2.497 [1.277–4.883]	0.007
Dominant	2.303 [1.414–3.752]	0.001	2.012 [1.200–3.373]	0.008
Recessive	0.579 [0.333–1.008]	0.054	0.610 [0.337–1.104]	0.103
MTHFR C677T				
CC	1.00		1.00	
CT	1.683 [0.990–2.860]	0.054	1.913 [1.085–3.375]	0.025
TT	2.511 [1.230–5.125]	0.011	3.117 [1.430–6.795]	0.004
Additive	2.493 [1.264–4.918]	0.008	2.936 [1.398–6.166]	0.004
Dominant	1.557 [0.980–2.575]	0.061	1.802 [1.094–2.968]	0.021
Recessive	0.468 [0.251–0.870]	0.017	0.437 [0.225–0.849]	0.015
Occupational stress				
Low	1.00		1.00	
Middle	1.300 [0.514–3.289]	0.580	0.753 [0.277–2.051]	0.579
High	2.740 [1.098–6.837]	0.031	1.629 [0.610–4.352]	0.330

### Effect of gene-gene and gene-occupational tension interactions on hypertension

**Figure 1:**
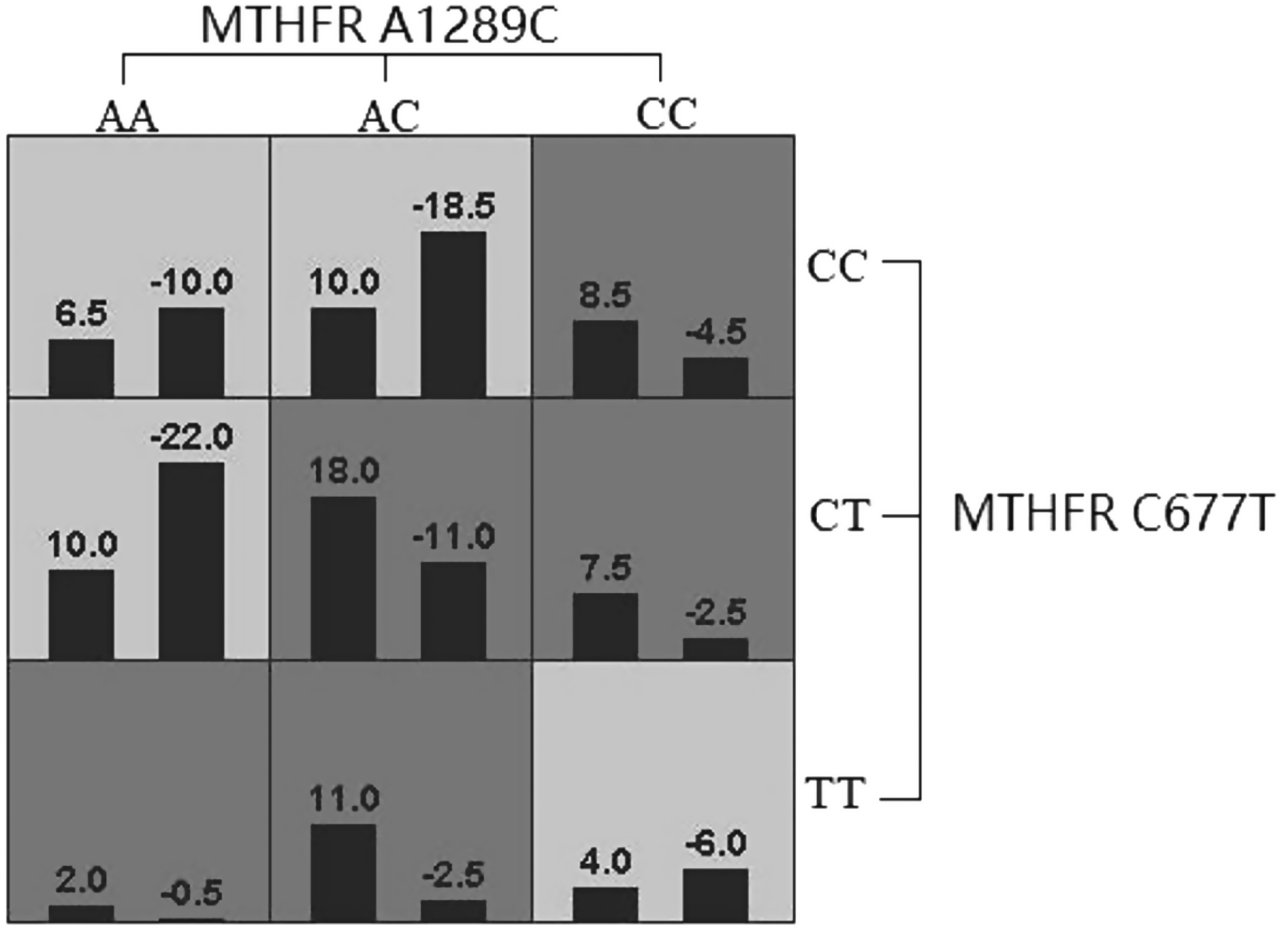
Forest plot of logistic regression analysis of the influence of genes and occupational stress.

**Figure 2:**
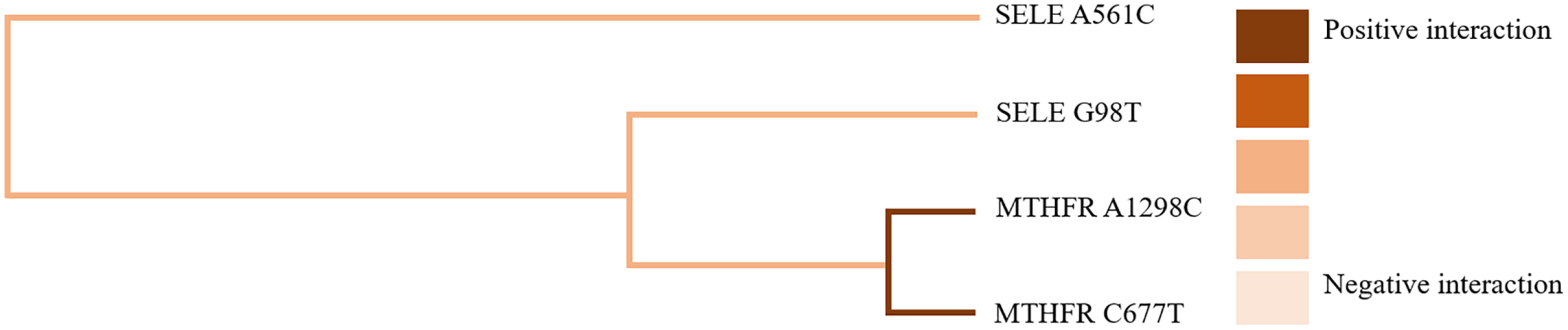
Gene-gene interaction model of MTHFR gene A1298C and C677T sites.

**Figure 3:**
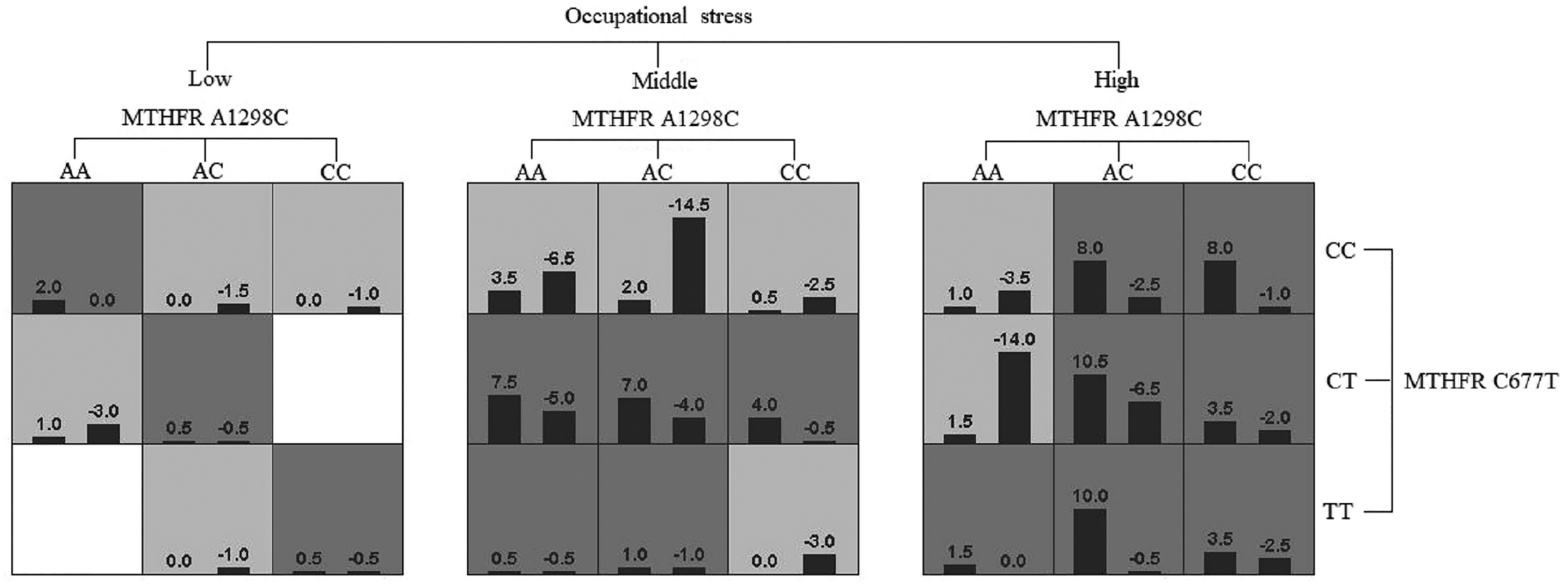
Gene-gene interaction dendrogram of MTHFR gene A1298C and C677T sites.

**Figure 4:**
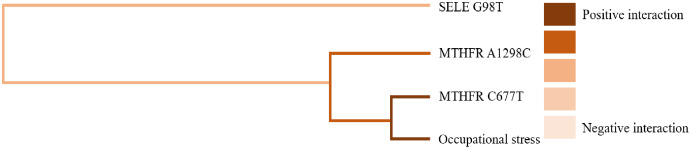
Gene-environment interaction model of MTHFR gene A1298C and C677T sites and occupational stress.

GMDR software was used to analyze the effects of gene-gene and gene-occupational tension interactions on hypertension. The gene-gene interaction showed the best interaction model between the MTHFR gene A1298C and C677T loci with a training set precision of 0.6677 and a test set precision of 0.6639, a sign test *P* = 0.001, and a cross-validation agreement coefficient of 10/10, as shown in Table 5 and Fig. 1. The dendrogram showed a strong positive interaction between the MTHFR gene A1298C polymorphism and the C677T loci, see Fig. 2. The gene-occupational tension interaction showed the best interaction model between MTHFR gene A1298C polymorphism, C677T polymorphism and occupational tension with a training set precision of 0.7662, test set precision of 0.7440, sign test *P* = 0.001, and cross-validation consistency coefficient of 10/10, see Fig. 3. The dendrogram showed a strong positive interaction between the C677T polymorphism of the MTHFR gene and occupational tension, see Fig. 4.

**Table 5:** GMDR model of the influence of gene-gene, gene-occupational stress interaction on the prevalence of hypertension.

Interaction model	Accuracy		Sign test (*P*)	CV consistency
	Training set	Testing set		
Gene-Gene				
A1298C	0.5903	0.5851	9 (0.0107)	10/10
A1298C*C677T	0.6677	0.6639	10 (0.0010)	10/10
A1298C*C677T*A561C	0.6929	0.6542	10 (0.0010)	7/10
A1298C*C677T*A561C*G98T	0.7310	0.6340	10 (0.0010)	10/10
Gene-Occupational stress				
Occupational stress	0.5993	0.5503	8 (0.0547)	7/10
Occupational stress*C677T	0.7010	0.6586	10 (0.0010)	9/10
Occupational stress*C677T*A1298C	0.7662	0.7440	10 (0.0010)	10/10
Occupational stress*C677T*A1298C*G98T	0.7958	0.7351	10 (0.0010)	10/10
Occupational stress*C677T*A1298C*G98T*A561C	0.8235	0.6916	10 (0.0010)	10/10

## Discussion

Hypertension is a major risk factor for cardiovascular disease and has a low control rate due to its complex and diverse etiology (Gheorghe et al., 2018). Many researchers have done numerous studies on environmental and genetic aspects and found that occupational stress is one of the risk factors for hypertension, but MTHFR gene and SELE base polymorphisms may lead to different levels of individual susceptibility to hypertension depending on geographical regions and ethnicity. Previous studies have also suggested the utility of hypertension susceptibility genotypes in Mendelian randomization (Fu et al., 2019). However, it has been argued that a single genotype in the MTHFR gene and SELE gene should not be used as independent markers in a Mendelian randomization design, but rather the study should be expanded to include gene-environment interactions. Therefore, this study not only explored the effect of genes, but also the effect of gene-environment interactions on hypertension.

Studies about some populations in the three northeastern provinces of China have found that polymorphisms in the MTHFR genes A1298C and C677T are not significantly associated with hypertension (Liu et al., 2019). In this case-control study of oil workers in Karamay, Xinjiang, adjusting for confounding factors, mutant genes at the A1298C polymorphism and the C677T polymorphism of the MTHFR gene were found to be susceptible genotypes for hypertension, which is consistent with the findings of and Alghasham et al. (2012). The human MTHFR gene is located on chromosome 1 (1P36.3) and has 11 exons, and the SNP sites that have a greater impact on enzyme activity are found at the C677T and A1298C loci, which are in the coding region.

The C677T polymorphism is a point mutation at the position 677 on MTHFR gene with the substitution of cysteine to thymine nucleotide at that position. This point mutation causes the substitution of alanine to valine in the MTHFR enzyme. The single nucleotide polymorphism of this gene reduces the thermostability of the MTHFR enzyme due to the decreased activity of the enzyme at 37 °C or higher. MTHFR enzyme activity in homozygous subjects is 50–60% lower at 37 °C and 65% lower at 46 °C compared with normal non-mutated controls (Rosenberg et al., 2019). The inability of the MTHFR enzyme to catalyse the conversion of 5,10-methylenetetrahydrofolate to 5-methyltetrahydrofolate leads to the rise of plasma homocysteine levels in the homozygous mutated subjects. Some researchers postulated that homocysteine could cause atherogenesis and thrombogenesis leading to substantialfibrosis and muscle cell hyperplasia,which in turn can be a risk factor for coronary artery disease (Stanger et al., 2004). In addition, The MTHFR C677T polymorphism was also reported to be associated with increased risk of myocardial infarction in young/middle-aged Caucasians. Individuals with the MTHFR 677TT genotype and a low folate status had a significantly higher risk of coronary heart disease. A recent study also reported the association between MTHFR C677T gene polymorphism and essential hypertension which is closely related to the increased level of Hcy (Ren, He & Cao, 2018).

The present study also yielded such results. Numerous studies have confirmed that homocysteine (HCY) is an independent risk factor for cardiovascular disease and can cause vascular lesions in the body, and HCY accumulation caused by MTHFR mutations may increase the risk of vascular complications in hypertensive patients. Scholars have demonstrated that HCY levels are significantly elevated in individuals carrying the 677TT genotype, suggesting that the mechanism of MTHFR C677T and the development of hypertension maybe related to abnormal HCY metabolism due to decreased mutant enzyme activity, which impairs the normal biological function of endothelial cells, thereby inducing oxidative stress that exacerbates the inflammatory response and puts the blood vessels in a persistent inflammatory state, etc. It has been suggested that the A1298C mutation does not inherently affect blood pressure levels but may play a regulatory role when individuals are accompanied by the MTHFR C677T mutation. In contrast, the present study showed that the mutant gene at the A1298C polymorphism of the MTHFR gene is a susceptible genotype for hypertension, which may be due to different geographical and ethnic differences.

When the A-C mutation occurs in the A1298C polymorphism, the transcriptionally expressed glutamate will be converted to alanine, which will affect the catalytic regulation of the enzyme. A case-control study found the A1298 C polymorphisms were related to depression severity (Chen & Chang, 2021). It has been suggested that although the A1298C mutation itself does not affect blood pressure levels, it may play a regulatory role when individuals are accompanied by the MTHFR C677T mutation. In contrast, the present study showed that the mutated gene with the A1298C polymorphism in the MTHFR gene is a susceptible genotype for hypertension, which may be due to differences across geography and ethnicity. The difference in the association of C677T and A1298C with hypertension may be due to their gene polymorphism, with the C677T mutation site present in the MTHFR gene The C677T mutation site is found in exon 4 of the MTHFR gene and is involved in encoding the catalytic structural domain of the N-terminal part of the enzyme, which directly affects the catalytic activity of the enzyme, whereas the A1298C mutation site is found in exon 7 of the MTHFR gene and encodes the C-terminal regulatory domain.

The study also found that the mutant gene at the G98T polymorphism of the SELE gene was the susceptible genotype for hypertension, which is consistent with the study of *Yesheng* Wei et al. (2003). The human SELE polymorphism, located on the long arm of chromosome 1, is a 13-kb DNA sequence that is a vascular endothelial adhesion molecule and is a marker of vascular endothelial function. Activation of the endothelium promotes atherosclerosis reduces the elasticity of the arterial wall and alters the responsiveness of the endothelium to vascular stimuli, and endothelial dysfunction maybe a major cause of hypertension. The G98T polymorphism is a single nucleotide polymorphism in the coding region caused by the mutation of G to T in the 5′ untranslated region of exon 2 of the E-selectin gene at the site 98 (98bp downstream of the transcription start site). It was found that T allele carriers are at high risk of hypertension and have high blood pressure values. The G98T polymorphism mutation can enhance the expressed SELE function by affecting the E-selectin 5’ untranslated region structure, thus causing enhanced adhesion, which in turn causes an inflammatory response, damage to endothelial cells, inhibition of NO and prostaglandin production, and diminished vascular responsiveness to endothelium-dependent vasotransfer substances, resulting in increased vascular resistance and, consequently, hypertension (Srivastava et al., 2018). There are fewer studies on SELE gene polymorphisms in other diseases. The SELE gene also appears to be associated with atherosclerosis, with monocytes adhering to vascular endothelial cells and migrating into the endothelium to take up lipids and transform into foam cells as an early event in the formation of atherosclerosis. Early events in the formation of atherosclerosis. Whereas there is no association between A561C gene polymorphism and hypertension. The differences between these studies may be due to epigenetic mechanisms involved in gene expression influenced by environmental conditions (*e.g*., lifestyle and diet).

A meta-study in a population of pregnant women found that the risk of hypertension was significantly increased in the presence of high air pollution and mutations in the MTHFR gene (Yang, Yang & Shiao, 2018), and a study by Fu et al. (2018) in Chinese children confirmed that there was also an interaction between the MTHFR gene and the effect of obesity on hypertension. Wu et al. (2015) showed that the interaction between internal environmental factors may be a potential factor contributing to elevated blood pressure. In this study, we used GMDR software to construct risk models for the effects of gene-gene and gene-environment associations on hypertension, and further analysis of the interactions between occupational stress and the MTHFR and SELE genes found strong positive between the A1298C polymorphism of the MTHFR gene and the C677T polymorphism, and between the C677T polymorphism of the MTHFR gene and occupational stress. Taylor et al. (2010) did interaction validation of different genes with the environment and came to similar conclusions. The influence of genes on the pathogenesis of hypertension may be the increased deposition of extracellular matrix components and their altered structure or cell-extracellular matrix attachment, which causes structural changes in the arterial wall. In contrast, the MTHFR gene is an important enzyme for folate metabolism, and mutations in the MTHFR gene cause hyperhomocysteinemia and homocystinuria, indirectly affecting changes in human blood pressure (Bayramoglu et al., 2015). SELE gene, on the other hand, plays a key role in the binding of lymphocytes and monocytes to endothelial cells. Its genetic polymorphisms may upregulate gene expression levels and thus affect the biological functions of its proteins. Several lines of evidence have shown that A561C and G98T gene polymorphisms can lead to a significant increase in blood pressure (Faruque et al., 2011). The special nature of oilfield operations, which often involve shift work, job evaluation, and learning new technologies lead to the occurrence of occupational stress as oilfield workers are often faced with loneliness, insomnia, and depression separated from their families (Yong et al., 2020). When both genetic and environmental factors are present, the risk of developing hypertension increases significantly.

## Conclusions

In conclusion, the risk of hypertension among oil workers in Karamay, Xinjiang, maybe influenced not only by environmental but also by genetic factors, and there is a strong interaction between the two, which provides an updated vision for the prevention and control of hypertension. This study demonstrated a strong interaction between gene-gene and gene-environment effects on hypertension, but there are several potential limitations that should not be overlooked: first, the sample size was small and there may be selection bias. Second, the genotyping method used in this study may have potential bias, and there are reports demonstrating higher sensitivity and accuracy of genotyping methods such as Mass Array and gene chips. Therefore, further sample expansion and selection of more appropriate genotyping methods must be performed in the future to confirm the results we observed in this study.

## Supplemental Information

10.7717/peerj.12914/supp-1 Supplemental Information 1 Data.

10.7717/peerj.12914/supp-2 Supplemental Information 2 Data introduction.

## Additional Information and Declarations

### Data Availability

The following information was supplied regarding data availability: The raw measurements are available in the Supplemental Files.

## References

[ref-21] Alghasham A, Settin AA, Ali A, Dowaidar M, Ismail H (2012). Association of MTHFR C677T and A1298C gene polymorphisms with hypertension. International Journal of Health Sciences.

[ref-9] Amenyah SD, Ward M, McMahon A, Deane J, McNulty H, Hughes C, Strain JJ, Horigan G, Purvis J, Walsh CP, Lees-Murdock DJ (2020). DNA methylation of hypertension-related genes and effect of riboflavin supplementation in adults stratified by genotype for the MTHFR C677T polymorphism. International of Journal Cardiology.

[ref-11] Amrani-Midoun A, Kiando SR, Treard C, Jeunemaitre X, Bouatia-Naji N (2016). The relationship between MTHFR C677T gene polymorphism and essential hypertension in a sample of an Algerian population of Oran city. International of Journal Cardiology.

[ref-33] Bayramoglu A, Urhan Kucuk M, Guler HI, Abaci O, Kucukkaya Y, Colak E (2015). Is there any genetic predisposition of MMP-9 gene C1562T and MTHFR gene C677T polymorphisms with essential hypertension?. Cytotechnology.

[ref-14] Chen HL, Hua Q, Guo JC, Xu J, Yang RQ (2008). Correlation between polymorphism of E-selection G98T and blood pressure in patient with essential hypertension. Journal of Cardiovascular and Pulmonary Diseases.

[ref-25] Chen DD, Chang R (2021). Associations of Hcy and the distribution of MTHFR A1298C with essential hypertension in Qinghai Tibetans. Chinese Journal of Clinical Research.

[ref-1] GBD 2019 Risk Factors Collaborators (2019). Global burden of 87 risk factors in 204 countries and territories, 1990–2019: a systematic analysis for the Global Burden of Disease Study 2019. The Lancet.

[ref-18] Gheorghe A, Griffiths U, Murphy A, Legido-Quigley H, Lamptey P, Perel P (2018). The economic burden of cardiovascular disease and hypertension in low- and middle-income countries: a systematic review. BMC Public Health.

[ref-12] Ghogomu SM, Ngolle NE, Mouliom RN, Asa BF (2016). Association between the MTHFR C677T gene polymorphism and essential hypertension in South West Cameroon. Genetics and Molecular Research.

[ref-34] Faruque MU, Chen G, Doumatey A, Huang H, Zhou J, Dunston GM, Rotimi CN, Adeyemo AA (2011). Association of ATP1B1, RGS5 and SELE polymorphisms with hypertension and blood pressure in African-Americans. Journal of Hypertension.

[ref-30] Fu L, Zhang M, Hu YQ, Zhao X, Cheng H, Hou D, Mi J (2018). Gene-gene interactions and associations of six hypertension related single nucleotide polymorphisms with obesity risk in a Chinese children population. Gene.

[ref-19] Fu L, Li YN, Luo D, Deng S, Wu B, Hu YQ (2019). Evidence on the causal link between homocysteine and hypertension from a meta-analysis of 40173 individuals implementing Mendelian randomization. The Journal of Clinical Hypertension.

[ref-3] Huang L, Lian QL, Zhen L, Wei DQ, Wang ZJ (2019). The influence of interaction between gene and environmental factors on the prevalence of hypertension for the elderly. Strait Journal of Preventive Medicine.

[ref-10] Ji Y, Yiorkas AM, Frau F, Mook-Kanamori D, Staiger H, Thomas EL, Atabaki-Pasdar N, Campbell A, Tyrrell J, Jones SE, Beaumont RN, Wood AR, Tuke MA, Ruth KS, Mahajan A, Murray A, Freathy RM, Weedon MN, Hattersley AT, Hayward C, Machann J, Häring HU, Franks P, de Mutsert R, Pearson E, Stefan N, Frayling TM, Allebrandt KV, Bell JD, Blakemore AI, Yaghootkar H (2019). Genome -wide and abdominal MRI data provide evidence that a genetically determined favorable adiposity phenotype is characterized by lower ectopic liver fat and lower risk of type 2 diabetes, heart disease, and hypertension. Diabetes.

[ref-4] Kokubo Y, Padmanabhan S, Iwashima Y, Yamagishi K, Goto A (2019). Gene and environmental interactions according to the components of lifestyle modifications in hypertension guidelines. Environmental Health and Preventive Medicine.

[ref-7] Liao B, Chen K, Xiong W, Chen R, Mai A, Xu Z, Dong S (2016). Relationship of SELE A561C and G98T variants with the susceptibility to CAD. Medicine.

[ref-2] Lu JP, Lu Y, Wang XC, Li XY, Linderman GC, Wu CQ, Cheng XY, Mu L, Zhang HB, Liu JM, Su M, Zhao HY, Spatz ES, Spertus JA, Masoudi FA, Krumholz HM, Jiang LX (2017). Prevalence, awareness, treatment, and control of hypertension in China: data from 1.7 million adults in a population-based screening study (China PEACE Million Persons Project). The Lancet.

[ref-8] Lu X, Wang L, Lin X, Huang J, Charles Gu C, He M, Shen H, He J, Zhu J, Li H, Hixson JE, Wu T, Dai J, Lu L, Shen C, Chen S, He L, Mo Z, Hao Y, Mo X, Yang X, Li J, Cao J, Chen J, Fan Z, Li Y, Zhao L, Li H, Lu F, Yao C, Yu L, Xu L, Mu J, Wu X, Deng Y, Hu D, Zhang W, Ji X, Guo D, Guo Z, Zhou Z, Yang Z, Wang R, Yang J, Zhou X, Yan W, Sun N, Gao P, Gu D (2015). Genome-wide association study in Chinese identifies novel loci for blood pressure and hypertension. Human Molecular Genetics.

[ref-5] Liu F, He J, Gu D, Rao DC, Huang J, Hixson JE, Jaquish CE, Chen J, Li C, Yang X, Li J, Rice TK, Shimmin LC, Kelly TN (2015). Associations of endothelial system genes with blood pressure changes and hypertension incidence: the gensalt study. American Journal of Hypertension.

[ref-20] Liu S, Liu M, Li Q, Liu X, Wang Y, Mambiya M, Zhang K, Yang L, Zhang Q, Shang M, Zeng F, Nie F, Liu W (2019). Association of single nucleotide polymorphisms of MTHFR, TCN2, RNF213 with susceptibility to hypertension and blood pressure. Bioscience Reports.

[ref-17] Liu LS (2019). 2018 Chinese guidelines for the management of hypertension. Chinese Journal of Cardiovascular Medicine.

[ref-6] McNulty H, Strain JJ, Hughes CF, Ward M (2017). Riboflavin, MTHFR genotype and blood pressure: a personalized approach to prevention and treatment of hypertension. Molecular Aspects of Medicine.

[ref-13] Ouyang Y, Wu H, Tan A, Yang H, Gao Y, Li H, Lu S, Hu Y, Tang X, Zhang H (2015). E-selectin gene polymorphism (A561C) and essential hypertension, Meta-analysis in the Chinese population. Herz.

[ref-24] Ren Y, He YH, Cao MJ (2018). Correlation analysis of MTHFR andMTRR gene related mutations with H type hypertension. J. Yan’an U.

[ref-22] Rosenberg N, Murata M, Ikeda Y, Opare-Sem O, Zivelin A, Geffen E, Seligsohn U (2019). The frequent 5,10-methylenetetrahydrofolate reductase C677T polymorphism is associated with a common haplotype in whites, Japanese, and Africans. The American Journal of Human Genetics.

[ref-23] Stanger O, Herrmann W, Pietrzik K, Fowler B, Geisel J, Dierkes J, Weger M (2004). Clinical use and rational management of homocysteine, folic acid, and B vitamins in cardiovascular and thrombotic diseases. Zeitschrift fur Kardiologie.

[ref-28] Srivastava K, Chandra S, Narang R, Bhatia J, Saluja D (2018). E-selectin gene in essential hypertension: a case-control study. European Journal of Clinical Investigation.

[ref-15] Tao N, Ge H, Wu W, An H, Liu J, Xu X (2018). Association of glucocorticoid receptor gene polymorphism and occupational stress with hypertension in desert petroleum workers in Xinjiang, China. BMC Medical Genetics.

[ref-32] Taylor JY, Sun YV, Hunt SC, Kardia SL (2010). Gene-environment interaction for hypertension among African American women across generations. Biological Research for Nursing.

[ref-26] Wei YS, Li Y, Zhang PG, Li XY, Huang CX (2003). Association of E-selectin exon 2 G98T gene polymorphism with hypertensive disease. Shandong Medical Journal.

[ref-31] Wu CY, Zhou XM, Dong M, Huang KQ, Xu H, Su Y, Wang L, Chen XG, Zhang HM, Dong CW (2015). Correlation between AT1R gene polymorphism and environmental factors ang its implication for TCM patterns in essential hypertension. Journal of Beijing University of Traditional Chinese Medicine.

[ref-16] Xing HY, Liu Y, Zhao A, Zheng W, Li T, Zheng YD, Wang Y, Zhang YM (2018). Effects of smoking, drinking and dietary factors on hypertension in subjects with hyperuricemia. Chinese Journal of Disease Control & Prevention.

[ref-29] Yang YL, Yang HL, Shiao SPK (2018). Meta-prediction of MTHFR gene polymorphisms and air pollution on the risk of hypertensive disorders in pregnancy worldwide. International Journal of Environmental Research Public Health.

[ref-35] Yong X, Gao X, Zhang Z, Ge H, Sun X, Ma X, Liu J (2020). Associations of occupational stress with job burn-out, depression and hypertension in coal miners of Xinjiang, China: a cross-sectional study. BMJ Open.

