# Supplementary material for: Effect of interaction between occupational stress and polymorphisms of MTHFR gene and SELE gene on hypertension
Source: PeerJ. 2022 Feb 16;10:e12914. doi: 10.7717/peerj.12914 (PMC8858580; doi:10.7717/peerj.12914)
Supplement: Supplemental Information 2 [file peerj-10-12914-s002.docx]

**Data-introduction**

Gender：male=1，female=2

Age：＜30=1,30~45=2，＞45=3

Working age：≤15=1，大于15=2

Educational level：High school or below=1，College or above =2

Marital status：Single=1，Married=2，Divorced/others=3

Income：≤5000=1，＞0500=2

Professional title：Junior or below=1，Intermediate or above=2

Shift work：No=1，Yes=2

Smoking：Yes=1，No=0

Drinking alcohol：Yes=1，No=0

BMI：18.5~24=1，﹥24=2

A1298C2：AA=0，AC=1，CC=2

A561C2：AA=0，AC=1，CC=2

G98T2：GG=0，GT=1，TT=2

C677T2：CC=0，CT=1，TT=2

Occupational stress：Low=1，Middle=2，High=3

Interaction：

A1298C：AA=0，AC+CC=1

A561C:AA=0，AC+CC=1

G98T:GG=0，GT+TT=1

C677T:CC=0，CT+TT=1
